# Supplementary material for: Perceived stress and associated factors among health care professionals working in the context of COVID-19 pandemic in public health institutions of southern Ethiopia 2020
Source: PLoS One. 2021 Jun 10;16(6):e0252809. doi: 10.1371/journal.pone.0252809 (PMC8191883; doi:10.1371/journal.pone.0252809)
Supplement: S1 File — (DOCX) [file pone.0252809.s001.docx]

**Questionnaire**

CODE______________________ Date ______________ Name of Facility______________

| **Socio-Demographic and Medical History** | | | | | |
| --- | --- | --- | --- | --- | --- |
| SN | Questions | Response Category | | Skip Pattern | |
|  | Sex | 1. Male 2. Female | |  | |
|  | Age | _______________ | |  | |
|  | Marital Status | 1. Married 2. Single 3. Divorced 4. Separated 5. Cohabitation 6. Widowed | |  | |
|  | Do you have children? | 1. Yes (Specify Number_____) 2. No | |  | |
|  | Residence (Place you are currently living in) | ___________ | |  | |
|  | With whom are you living? | 1. Alone 2. Spouse 3. Parents 4. Friends 5. Other (Specify)------------- | |  | |
|  | Profession | 1. Medical Doctor 2. Clinical Nurse 3. Midwifery 4. Public Health Officer 5. Medical Laboratory Technician/Technologist 6. Pharmacist 7. Anesthetist | |  | |
|  | Educational status | 1. Diploma 2. First degree 3. Second degree 4. Level 3 or 4 5. Other (specify)------------- | |  | |
|  | How long have you served? | Put work experience in years/ months-------___________ | |  | |
|  | Religion | 1. Orthodox 2. Protestant 3. Muslim 4. Catholic 5. Other (Specify) ________________ | |  | |
|  | Which Ward/case team/Department are you currently working in? | 1. Emergency Ward 2. OPD(including Para-Medical) 3. Medical Ward 4. Surgical(including OR) Ward 5. Pediatrics(including NICU) Ward 6. Maternity (Gyn-OB) 7. Laboratory 8. Regional Lab 9. Dispensary | |  | |
|  | Do you have any medical conditions/comorbidities like? | 1. DM (1. Yes 2. No) 2. HIV/AIDS (1. Yes 2. No) 3. HBV (1. Yes 2. No) 4. ASTHMA (1. Yes 2. No) 5. Prior Stress (1. Yes 2. No) 6. Prior Anxiety (1. Yes 2. No) 7. Other (Specify) ____________ | |  | |
| **Psycho-social context, Individual psychosocial factors and substance use** | | | | | |
| SN | Questions | | Response Category | | Skip Pattern |
|  | In your opinion, how are community members responding to prevention practices? | | 1. Not at all 2. Somewhat 3. Moderately 4. To a great extent | |  |
|  | Being part of the COVID-19 response, do you feel valued by   - 1. Your family? | | 1. Yes 2. No | |  |
|  | - 1. The surrounding community? | | 1. Yes 2. No | |  |
|  | Have you ever had Contact with COVID 19 confirmed or suspected individuals? | | 1. Yes 2. No | |  |
|  | Are Personal Protective Equipment adequately availability for your use? | | 1. Yes 2. No | |  |
|  | Have you ever taken COVID 19 related trainings so far? | | 1. Yes 2. No | |  |
|  | Do you have regular access to mainstream and social media? | | 1. Yes    1. Television    2. Radio    3. Telegram    4. Facebook    5. Other (Specify) 2. No | |  |
|  | Do you have line of Communication regarding COVID 19 updates? | | 1. Yes 2. No | |  |
|  | Does the government support your role in preventing and controlling COVID 19? | | 1. Not at all 2. Somewhat 3. Moderately 4. To a great extent | |  |
|  | How confident are you to coping with COVID 19 related stresses with your personal coping strategies? | | 1. Not at all 2. Somewhat 3. Moderately 4. To a great extent | |  |
|  | Do you feel you are susceptible to contracting COVID 19? | | 1. Yes 2. No | |  |
|  | Do you have any COVID 19 related worry? | | - 1. Yes   2. No | | If “No”, skip to question number 25 |
|  | What kind of COVID 19 related fear are you currently experiencing?  (Multiple responses are possible) | | 1. Fear of contracting the virus. 2. Fear of transmitting the virus to family members 3. Fear of being discriminated. 4. Fear of being abandoned by spouse or girlfriend/boyfriend. 5. Feeling of helplessness 6. Fear of isolation 7. Fear of death 8. Other, ___________ | |  |
|  | Do you believe that the suggested prevention and control practices are able to contain the spread of the pandemic? | | 1. Yes 2. No | |  |
|  | Do you drink alcohol? | | 1. Yes 2. No | |  |
|  | Do you smoke cigarette? | | 1. Yes 2. No | |  |
|  | Do you perform physical exercise? | | 1. Yes 2. No | |  |
|  | Do you have anyone whom you can trust and confide in? | | 1. Yes 2. No | |  |
|  | Do you get along well with people? | | 1. Yes 2. No | |  |
|  | Do you often feel overwhelmed by the demands of everyday life? | | 1. Yes 2. No | |  |
|  | Do you often feel you cannot make it? | | 1. Yes 2. No | |  |
|  | Do you tend to be influenced by people with strong opinions? | | 1. Yes 2. No | |  |
|  | Do you tend to worry about what other people think of you? | | 1. Yes 2. No | |  |

| **Psychological related Questions 3: Perceived stress scale** | | | | | | |
| --- | --- | --- | --- | --- | --- | --- |
| **Direction**: The questions in this scale ask you about your feelings and thoughts during the last month. In each case, you will be asked to indicate by circling *how often* you felt or thought a certain way.  **Never = 0 Almost never =1 Sometimes =2 Fairly often = 3 Very often=4** | | | | | | |
| SN | Questions | 0 | 1 | 2 | 3 | 4 |
| 1 | In the last month, how often have you been upset because of something that happened unexpectedly? |  |  |  |  |  |
| 2 | In the last month, how often have you felt that you were unable to control the  important things in your life? |  |  |  |  |  |
| 3 | In the last month, how often have you felt nervous and stressed? |  |  |  |  |  |
| 4 | In the last month, how often have you felt confident about your ability to handle your personal problems? |  |  |  |  |  |
| 5 | In the last month, how often have you felt that things were going your way? |  |  |  |  |  |
| 6 | In the last month, how often have you found that you could not cope with  all the things that you had to do? |  |  |  |  |  |
| 7 | In the last month, how often have you been able to control irritations in  your life? |  |  |  |  |  |
| 8 | In the last month, how often have you felt that you were on top of things? |  |  |  |  |  |
| 9 | In the last month, how often have you been angered because of things that  happened that were outside of your control? |  |  |  |  |  |
| 10 | In the last month, how often have you felt difficulties were piling up so high that  you could not overcome them? |  |  |  |  |  |
