# Supplementary material for: Perceived stress and associated factors among health care professionals working in the context of COVID-19 pandemic in public health institutions of southern Ethiopia 2020
Source: PLoS One. 2021 Jun 10;16(6):e0252809. doi: 10.1371/journal.pone.0252809 (PMC8191883; doi:10.1371/journal.pone.0252809)
Supplement: Attachment — Submitted filename: comments for author.docx [file pone.0252809.s003.docx]

1. Recommendation

Major Revision

2. Is the manuscript technically sound, and do the data support the conclusions?

Partly

3. Has the statistical analysis been performed appropriately and rigorously?

Yes

4. Does the manuscript adhere to the PLOS Data Policy?

Yes

5. Is the manuscript presented in an intelligible fashion and written in standard English?

Yes

6. Review Comments to the Author

- Exclusion criteria needs to be clarified further
- Who were your data collectors with their level of educational background? (number of data collectors and supervisors should be clarified)
- For how many days training was given for data collectors?
- Please rewrite your operational definitions
- In the category of professions, why you omitted professionals like mental health and environmental health workers?

7. Would you like your identity revealed to the authors of this submission?

No

8. Do you have any potentially competing interests?

None
