# Supplementary material for: Perceived stress and associated factors among health care professionals working in the context of COVID-19 pandemic in public health institutions of southern Ethiopia 2020
Source: PLoS One. 2021 Jun 10;16(6):e0252809. doi: 10.1371/journal.pone.0252809 (PMC8191883; doi:10.1371/journal.pone.0252809)
Supplement: Attachment — Submitted filename: point by point response stress.docx [file pone.0252809.s004.docx]

**Author’s Point-by-Point Response to the Reviewer's and Editors Reports**

**Title**: Perceived stress and associated factors among health care professionals working during COVID-19 pandemic in public health institutions of southern Ethiopia 2020

**Corresponding author**: Asmare Getie/ asmaregetie2017@gmail.com

Authors:

Asmare Getie

ID: - PONE-D-20-38452

Journal: PLOS ONE
**Point by point response to Reviewers and Editors**

First of all, the authors would like to thank PLOSE ONE Journal editors and the respective reviewers for reviewing our manuscript and providing the necessary comments to be corrected. As per the comments given, we have made corrections point by point to comment. The authors tried to answer all the issues raised by editorial team and reviewers. Please note that we gave our response in blue font color.

**Point by point response for Editors**

Question 1: The authors did not provide any cut-off value for perceived stress in their operational definitions making the interpretation of the data very difficult. It is not helpful to present a percentage of perceived stress when there are no established cut-offs.

Response 1: Thank you for your nice and constructive suggestions: we have used a total score of >20 points was considered as the cut off for experiencing perceived stress based on perceived stress scale.

**Point by point response for Reviewers**

Question 1: The study is about perceived stress of healthcare workers in the context of COVID-19. May the authors include “in the context of…” instead of “during” in the title?

Response 1: Thank you for your constructive comment. We have corrected accordingly as, Perceived stress and associated factors among health care professionals working in the context of COVID-19 pandemic in public health institutions of southern Ethiopia 2020

Question 2: There are editorial problems.

Response 2: thank you, the editorial problems were corrected, you can appreciate it in the track change and clean manuscript.

Question 3: Why written consent was not obtained from the participants?

Response 3: during the data collection the transmission of COVID-19 and the fear related with COVID-19 transmission was high. And the main route of transmission was by body contact and sharing of equipment’s. to Avoid the risk of transmission the data were collected using KOBO collector software, and the researchers decided to use verbal consent.

Question 4: In the methods section of the abstract, it says: “The study participants were selected using simple random sampling technique after allocating a proportion to each health institute based on the size of HCPS. A pretested and structured interviewer-administered questionnaire using KOBO collect survey tool was used to collect data”. What are HCPS and KOBO? This should be fully spelled in first use.

Response 4: Thank you for your nice suggestion, HCPS mean that health care professionals and it was written based on your suggestion, and KOBO collection survey is based on the open source Collect app by get ODK and is used for primary data collection in humanitarian emergencies and other challenging field environments. With this app you enter data from interviews or other primary data -- online or offline. There are no limits on the number of forms, questions, or submissions (including photos and other media) that can be saved on your device

Question 5: The tools of data collection, at least for main outcomes, are not briefly stated in the abstract. May the authors also state the cut off of dichotomizing the outcome measures in this abstract section?

Response 5: thank you for this nice comment and suggestion, the cutoff point was stated in the abstract part as well as in the operational definitions.

Question 6: In the conclusion section of the abstract, the authors have repeated the findings than showing the readers about the implications of the findings. Indeed, the first couple of sentences look non-relevant to the conclusion.

Response 6: thank you, it was amended as per recommendation. The irrelevant sentence was omitted.

Question 7: Introduction on page 4, line 83-84, it says: “There is a dearth of information regarding stress related to COVID-19 in Ethiopia”. What about similar study conducted in Dilla, southern Ethiopia? What about green literature in this area in Ethiopia?

Response 7: a similar study that was conducted in Dilla southern Ethiopia was not was not addressed different health institutions and all relevant data cannot be founded there. Therefore scarcity of data regarding COVID-19 in the southern region was critical.

Question 8: The authors used simple random sampling. There is also a statement about multistage sampling? If multistage sampling was used, was it stratified by the types of institutions: hospital, HC or health posts?

Response 8: stratification was not used. Just we had proportionally allocated to each health institution based on the number of health care professionals and we have used simple random sampling technique to select study subjects from the selected health institutions.

Question 9: KOBO” requires further description. The outcome measures and their psychometric properties including the issues of validity in the Ethiopian context is not given. Please, do it. The cut off values in the original tools and in the current manuscript are not given. Otherwise it is difficult to interpret the findings and there is no gauge.

Response 9: KOBO collection survey is based on the open source Collect app by get ODK and is used for primary data collection in humanitarian emergencies and other challenging field environments. With this app you enter data from interviews or other primary data -- online or offline. There are no limits on the number of forms, questions, or submissions (including photos and other media) that can be saved on your device.

The outcome measures were validated in Ethiopian contexts, different studies in Ethiopia have used this tool to measure perceived stress, so the issue of validity was not a concern. The cutoff value was stated in operational definition, those who scored greater than 20 from the total 40 score were considered as they had perceived stress.

Question 10: With respect to the data quality, how the data was entered. Would you describe it?

Response 10: The data were collected using KOBO collection survey which was uploaded on smart phone, after the data was collected it was sent to the server and which was presented as EXCEL sheet. Finally the data was exported from EXCEL sheet in to SPSS version 25 for processing and analysis.

Question 11: The Ethiopian population is more than 80% rural. But, the samples indicated that only 12% rural participants. This indicates non-random sampling??

Response 11: the study was conducted on health care professionals who were working in different health institutions and most of health institutions were available in the town starting from woreda level, because electric city is a minimum requirement for health institutions and in the rural environment the access was almost null. Therefore even if some of the study participants were born and grow in the rural community, currently they are working in health institution that were available at woreda level, special woreda and zonal level. So the respondents might be reported their residence as urban, since currently they are living there.

Question 12: Many sentences are not clear in the results. ex “From the total respondents more than half 409(51.3%) of health care professional’s responded that the community response to COVID -19 as somewhat followed by 247(31%) not at all”.

Response 12: Thank you for your nice comment, community response to COVID -19 (to what extent the community was given a response to COVID-19) is one variable which was assessed. There were four options for this variables, to a great extent, moderately, somewhat and not all. In the result part nearly more than half were responded as the community response to COVID-19 was somewhat and followed by 247 (31%) not at all.

Question 13: I could not follow what Table 2 is presenting? The use of language and given options for the items are not clear.

Response 13: we had made some modification on the language, you can appreciate it from the manuscript, and the options were used from validated and contextualized tools that were used in other previous studies.

Question14: The following variables are not clear
a. Government support: government support is similar for similar healthcare institutions. So why it is included as variable? Moreover, how it was measured?
b. Valued by community: what does it mean? How was it assessed?
c. Response of community members: what is it? How has it been assessed?
d. What was the assessment tool for “confidence in coping”?

Response 14: Government support might be similar for similar healthcare institutions, but it can be a variable, the intention of the researchers were to know the association of having or not having government support to healthcare institutions and perceived stress on health care professionals. Since it is a pandemic government support might have a significant effect on perceived stress of health care professionals.

Valued by community mean that, (does the community gave a value or recognition to your effort and work?). In the context of COVID-19 health care professionals were the frontline workers who were fighting against a pandemic to save the community and the community might have a value to health care professionals. The intention of the researchers were to know it the magnitude and its association with perceived stress. It was assessed by asking the respondents since they were living in the community, they can appreciated how the community gave value to their effort and respond it accordingly. Just by asking does the community give value for your effort to fight against COVID-19?

Response of community members mean that how the community give response to prevention and transmission of COVID 19? And there were four options: to a great extent, moderately, somewhat and not at all.

Confidence in coping stress was assessed by asking the respondents how much you are confident enough to coping stress. And it had four options: to a great extent, moderately, somewhat and not at all.

Question 15: Conclusion
“Lack of line of commutation to providing up to date information, lack of confidence on coping stress and not getting along well with individuals are factors associated with developing perceived stress”. What does it mean?

Response 15: lack of line of communication to providing up-to-date information about COVID-19, mean that lack of updated information regarding COVID-19, it was stastically significant to perceived stress. Lack of confidence in coping stress mean that the respondents were not confident to cope stressful situations and any pandemic can create a stress full situation and there was significantly association between lack of confidence in coping stress and perceived stress.

Response to reviewer 2

Question 1: Exclusion criteria needs to be clarified further
who were your data collectors with their level of educational background? (Number of data collectors and supervisors should be clarified)
For how many days training was given for data collectors?
Please rewrite your operational definitions
In the category of professions why you omitted professionals like mental health and environmental health workers?

Response 1: thank you very much for your nice suggestion, the exclusion criteria was amended accordingly in the manuscript, data collectors and supervisors including their level of educational back ground, duration of trainings were incorporated in the manuscript.

Operational definitions was rewrite again

Majority of health institutions had no mental health and environmental health workers that is why the category omitted this professionals.
