# Supplementary material for: Perceived stress and associated factors among health care professionals working in the context of COVID-19 pandemic in public health institutions of southern Ethiopia 2020
Source: PLoS One. 2021 Jun 10;16(6):e0252809. doi: 10.1371/journal.pone.0252809 (PMC8191883; doi:10.1371/journal.pone.0252809)
Supplement: Attachment — Submitted filename: comments.docx [file pone.0252809.s005.docx]

1. Recommendation

Accept

2. Is the manuscript technically sound, and do the data support the conclusions?

Yes

3. Has the statistical analysis been performed appropriately and rigorously?

Yes

4. Does the manuscript adhere to the PLOS Data Policy?

Yes

5. Is the manuscript presented in an intelligible fashion and written in Standard English?

Yes

6. Review Comments to the Author

All comments were addressed

7. Would you like your identity revealed to the authors of this submission?

No

8. Do you have any potentially competing interests?

None
